# Behavioural and transcriptional changes in post-mating females of an egg parasitoid wasp species

**DOI:** 10.1098/rsos.181453

**Published:** 2019-01-23

**Authors:** Peng-Cheng Liu, De-Jun Hao

**Affiliations:** 1 Co-Innovation Center for Sustainable Forestry in Southern China, Nanjing Forestry University, Nanjing City, Jiangsu Province, People's Republic of China; 2 College of Forestry, Nanjing Forestry University, Nanjing City, Jiangsu Province, People's Republic of China

**Keywords:** mating, parasitoid wasp, transcriptional analysis, post-mating behaviour

## Abstract

In many animals, mating is essential for the production of offspring by females; however, mating seems to not be necessary in Hymenoptera insects. Virgin females can produce offspring, although the sex of the offspring is all male. Usually, behavioural and physiological changes are induced by mating in female insects, including parasitoid wasps. However, very little is known about the resulting changes in gene expression that contribute to the post-mating response in females; thus, we studied this aspect in the egg parasitoid wasp species *Anastatus disparis* (Hymenoptera: Eupelmidae) by transcriptional analysis. A total of 55 differentially expressed genes were identified in post-mating females, and most of the genes (90.9%) were downregulated. Upregulated genes encoded products that were mainly involved in fatty acid synthesis and pyrimidine metabolism, while the downregulated genes were mainly involved in substance transport and metabolism. In addition, post-mating *A. disparis* females exhibited a tendency to accelerate egg maturation and became unreceptive to further mating. Based on the transcriptional data, we discuss how specific genes mediate these behavioural and physiological changes. Overall, our study provided new and comprehensive insights into post-mating changes in females and provided a basis for future mechanistic studies.

## 1. Background

In many animals, mating is essential for the production of offspring by females [1,2]; however, mating seems to not be necessary in Hymenoptera, in which sex determination is haplodiploid. Usually, males develop from unfertilized eggs and are haploid, while females develop from fertilized eggs and are diploid. Thus, virgin females can produce offspring, but the sex of all these offspring is male [3,4]. Mating often induces behavioural and physiological changes in female insects [5], including parasitoid wasps. Most commonly, these changes include increased oviposition behaviour and repression of subsequent sexual activity as a result of the transfer of male accessory gland substances [6]. In addition, in Hymenoptera parasitoids, virgin and mated female wasps may behave differently because of differences in the benefits and costs of mating [1]. Compared to mated females, unmated females are usually likely to exhibit reduced fitness, especially in highly structured populations, where brothers compete for mates and the reproductive return through sons is low, requiring females to minimize the number of male offspring [7–9]. Unmated females can increase their fitness by producing only a few small sons, sufficient for mother–son mating, to produce female offspring [10,11]. In many species, mated females produce more offspring than virgin females [12–15]; however, there have been a few reports of virgin females producing more offspring than mated females [16–18], and many species exhibit no significant difference [1,19–23].

In addition, there have been several studies on the effect of female mating status on other aspects including superparasitism [24,25], host discrimination capacity [26], longevity [1,17,27], foraging [28] and offspring fitness [9]. Hypothetically, virgin females need to trade-off between either (1) searching for hosts and producing sons immediately or (2) searching for mates and perhaps producing both sons and daughters later in life [13,16,28,29]. Generally, studies on the difference between virgin and mated female parasitoid wasps have focused on behaviour and demographic parameters. However, very little is known about the resulting changes in gene expression in females that contribute to the post-mating response. Thus, we attempted to study the changes in gene expression in post-mating females in the egg parasitoid wasp species *Anastatus disparis* (Hymenoptera: Eupelmidae) by transcriptomic analysis.

*Anastatus disparis* is an egg parasitoid of several harmful species of Lepidoptera that are primarily considered forest pests in China [30]. In previous studies, *A. disparis* is considered a potential biological control agent for *Lymantria dispar* which is an important defoliator of broad-leaved and coniferous trees [30–32]. As with other parasitoids, the sex determination of *A. disparis* is haplodiploid; both virgin and mated females can produce several hundred offspring in a lifetime and live for more than a month in the wild [23,30]. In terms of oviposition and longevity, there are no significant differences between virgin and mated *A. disparis* females [23,30]. Here, we also focused on other reproductive aspects of changes induced by mating in *A. disparis* females (e.g. mating ability and egg load). Besides identifying changes in gene expression prompted by mating, our study attempted to provide new and comprehensive insights into post-mating changes in females and provide a basis for future mechanistic studies.

## 2. Material and methods

### 2.1. Insect cultures

*Anastatus disparis* colonies were first established from a population reared on an *L. dispar* egg mass collected in Longhua County, Hebei Province (41°31′ N, 117°74′ E) in March 2012, and the colony was subsequently maintained on *Antheraea pernyi* eggs. *Antheraea pernyi* is of commercial interest due to the use of its pupae in silk production. Pupae of *A. pernyi* were purchased from Qinhuangdao, Hebei Province, China. Adult *A. pernyi* emerged from the pupae at 25–30°C. Eggs of *A. pernyi* were obtained by dissecting adult female abdomens and maintained at 0°C [25,33]. Approximately 20 hosts were offered to a female for oviposition lasting 24 h at 26–28°C. Then, we isolated the hosts individually in polyethylene tubes (height: 7.5 cm; diameter: 1 cm) whose openings were covered with cotton balls to prevent any mating behaviour before the start of the experiment. The parasitized hosts were incubated at a temperature of 28 ± 0.5°C, a relative humidity of 70 ± 5% and a photoperiod of 14 L : 10 D. After approximately 18 days [23], females and males started to emerge and were collected daily. Before the experiment began, the adult wasps were fed honey water (honey : water = 4 : 6) on cotton balls [30,33].

### 2.2. Transcriptomic analyses

For the transcriptomic experiment, 2-day-old virgin and 2-day-old mated female adults were selected. Each treatment included three replicates, and each replicate included 15 adults (virgin or mated females). Similar to *Anastatus* sp. [30], most *A. disparis* adults emerge daily in the morning, especially from 9.00 to 10.00. These virgin females were collected during this period, then divided into two groups. One group of females was maintained in a virgin condition, while each female from the other group was offered one new emerged male, and the mated females who mated within 2 h were selected. At 12.00 on day 2, the whole bodies of adults in the same treatment were pooled into a plastic tube (1.5 ml), snap frozen in liquid nitrogen, and transferred to a −80°C freezer for long-term storage. RNA from each sample group was extracted with TRIzol reagent (Invitrogen, USA). A total of 3 µg of total RNA from each sample was converted into cDNA using the NEBNext^®^ Ultra^™^ RNA Library Prep Kit for Illumina^®^ (NEB, USA). In total, six cDNA libraries were constructed and subsequently sequenced with the Illumina HiSeq 2000 platform by Beijing Biomarker Technologies Co. Ltd, yielding raw reads. Raw sequence data generated were deposited into Sequence Read Archive database of NCBI with the accession no. PRJNA505044. Clean reads were obtained by removing reads containing the adapter sequence, poly-N reads and low-quality reads from the raw data using FASTX-Toolkit (http://hannonlab.cshl.edu/fastx_toolkit/), and these clean reads were used for further analysis. Then, all the high-quality reads from the six samples were pooled and assembled using Trinity software (v. 2.5.1) with the default parameters [34]. A tool of *TransRate* was used in our study to evaluate the transcriptome assembly [35]. We chose the longest isoform of each gene to construct the unigene set. After the isoforms were selected, these assembled transcripts were predicted to be the unigenes produced. Bowtie was used to align reads to unigenes [36], then identified putatively expressed genes by RSEM [37] using the reads per kb per million reads (RPKM) method. For functional annotation, the pooled assembled unigenes were searched using BLASTX (v. 2.2.31) against five public databases, namely, Swiss-Prot, euKaryotic Orthologous Groups (KOG), NCBI non-redundant protein sequences (nr), KEGG Ortholog database (KO) and Gene Ontology (GO), with an *E*-value cut-off of 10^−5^. Differentially expressed genes (DEGs) were indentified using DESeq2 package (v. 1.6.3) in R, and incorporate RSEM reads into DESeq2 using tximport [38]. Genes with at least a two-fold change (FC) (i.e. log_2_|FC| greater than or equal to 1) and a false discovery rate (FDR) less than 0.01 were considered to be differentially expressed. The GOseq R package [39] was used to implement the statistical enrichment of DEGs in the GO database, and an adjusted *p*-value < 0.05 was chosen as the significance cut-off.

### 2.3. Mating

All adults were collected from 9.00 to 11.00 every day. Then, a virgin female (1-day-old) was supplied with a newly emerged virgin male in a Petri dish (height: 1.5 cm; diameter: 8 cm) at an environmental temperature of 26 ± 1°C. We recorded whether and when the female exhibited mating behaviour over a period of 60 min. If multiple matings occurred in this period, we also recorded the mating times. Then, the mated females were selected and removed and then offered another newly emerged male for 60 min, and the condition of mating (e.g. whether and when mating behaviour was exhibited, and mating times) was examined. Additionally, females that mated on the first day were fed honey water (honey : water = 4 : 6) on cotton balls. On days 2, 3, 4 and 5 at 11.00, these females were supplied with newly emerged males to examine mating. As a control, 2-, 3-, 4- and 5-day-old virgins were also tested.

### 2.4. Quantitative real-time polymerase chain reaction

Mating generally causes changes in attractiveness, that is correlated with pheromone levels [40,41]. Many studies of lepidopteran species [42] and other insect orders [43,44] have shown that pheromone biosynthesis in females is stimulated by a brain factor known as pheromone biosynthesis-activating neuropeptide (PBAN). Therefore, we aimed to test whether a change in pheromone may result in mated females becoming less attractive by evaluating the mRNA expression of PBAN between mated female and virgin through quantitative real-time polymerase chain reaction (qRT-PCR) analysis. Total RNA was extracted from the whole bodies of mated female and virgin female adults using TRIzol (Invitrogen, USA) according to the manufacturer's protocols, and then resuspended in nuclease-free water; finally, the RNA concentration was measured using a Nanodrop (Thermo Scientific Nanodrop 2000; USA). Approximately 0.5 mg of total RNA was used as a template to synthesize the first-strand cDNA using a PrimeScript RT Reagent Kit (TaKaRa; Japan) following the manufacturer's protocols. The resultant cDNA was diluted to 0.1 mg ml^−1^ for further qRT-PCR analysis (ABI StepOne Plus; USA) using SYBR Green Real-Time PCR Master Mix (TaKaRa; Japan). qRT-PCR reaction was amplified with 2 µl of cDNA template, 10 µl of 2×SYBR Green Master Mix and 0.4 µl of each primer (10 µmol µl^−1^), to a final volume of 20 µl by adding water. The cycling parameters were 95°C for 30 s followed by 40 cycles of 95°C for 5 s and 62°C for 34 s, ending with a melting curve analysis (65°C to 95°C in increments of 0.5°C every 5 s) to check for nonspecific product amplification. Relative gene expression was calculated by the 2^−ΔΔCt^ method using the housekeeping gene translation elongation factor 1-alpha (EF1A) as a reference to eliminate sample-to-sample variations in the initial cDNA samples. Primers (table 1) for PBAN and EF1A gene were designed using Primer Express 2.0 software.

**Table 1.** Primer pairs used for expression analysis using qRT-PCR.

gene name	primer sequences
PBAN	forward: 5′-CGAAGCTCCGATGTTGAAGG-3′
	reverse: 5′-AGTCTTGGACCGAACCACAT-3′
EF1A	forward: 5′-ACCACGAAGCTCTCCAAGAA-3′
	reverse: 5′-AATCTGCAGCACCCTTAGGT-3′

### 2.5. Egg load determination

Unmated *A. disparis* females and females mated with conspecific males were dissected at ages ranging from 1 to 5 days for determination of egg loads. Egg loads were measured in terms of the number of mature eggs in the ovaries [45]. Unmated individuals that emerged each day from 9.00 to 11.00. were collected. To obtain mated females, newly emerged males were supplied to a virgin female (1-day-old) for mating, and mating behaviour was observed. All female adults were fed honey water (honey : water = 4 : 6) until dissection. The selected adults were subjected to sudden death at −80°C, and then the abdomens were placed into a Petri dish with a saline solution. We counted the number of mature eggs by dissecting the abdomens using forceps under a microscope (Leica M205A, Germany). In total, 15 replicates were performed for each treatment.

### 2.6. Statistical analysis

All analyses were performed using R software, version 2.14.1. The chi-square test was used to determine the effects of female age on the rate of mating. Prior to analysis, the raw data were tested for normality and homogeneity of variance with Kolmogorov–Smirnov and Levene's tests, respectively, and the data were transformed if necessary. The q-PCR data comparing gene expression in mated females and virgin were analysed with an independent *t*-test. In addition, a generalized linear mixed model (GLMM) was applied to test for the effects of mating status and female age on egg loads. For the analysis of GLMM, we used the lme4 package [46]. Egg loads were measured as response variables, with mating status and female age as fixed effects. Interactions are presented only where significant at a level of *p* < 0.01; this criterion for significance is recommended when testing interactions [47]. The positive/negative relationship between maternal age and egg load numbers was tested by correlation analysis.

## 3. Results

### 3.1. Transcriptomic analyses

We constructed six cDNA libraries derived from three *A. disparis* mated female and virgin adult samples. Approximately 8.57 Gb of paired-end reads were produced for each RNAseq sample. After removing reads containing adapter sequences, poly-N reads and low-quality reads from the raw data, approximately 7.17 Gb of clean reads were obtained from each sample. The percentages of Q30 were higher than 93.62% in each sample, which showed that sequencing of each sample was of high quality.

All high-quality reads from the six samples were pooled and assembled using Trinity with the default parameters, and the *TransRate* score of our assembly was 0.19 (optimized score of 0.23). A total of 132 543 transcripts with lengths longer than 300 bp were generated. More than half of the transcripts (73,211, 55.23%) were longer than 1 kb in length, whereas 44.76% (13 951) were between 300 and 1000 bp in length, and the N50 size was 5020 bp. Then, these assembled transcripts were predicted to be produced from a total of 57 152 unigenes. The N50 size of the unigenes was approximately 1935 bp, and their mean length was 1044.11 bp. 49.61% unigenes were between 300 and 500 bp in length, and half were longer than 500 bp (electronic supplementary material, table S1).

For annotation, the pooled assembled unigenes were searched using BLASTX against five public databases with an *E*-value cut-off of 10^−5^. A total of 28 174 unigenes were successfully annotated (table 2). Using our assembled transcriptome as a reference, we identified putatively expressed genes using the RPKM method, and genes with at least a two-FC and FDR less than 0.01 were defined as DEGs. Consequently, 55 DEGs were identified, including 5 upregulated and 50 downregulated genes in mated females (table 3). As shown in table 2, 12 genes were found in the GO database, 19 in KOG, 44 in nr, 25 in Swiss-Prot and 7 in KEGG. The upregulated genes after mating included those that encoded products that were mainly involved in fatty acid synthesis and pyrimidine metabolism. Downregulated genes were mainly involved in substance transport and metabolism (e.g. amino acids, carbohydrates and lipids).

**Table 2.** Functional annotation of assembled unigenes and differentially expressed genes (DEGs).

annotation database	annotated unigenes	number of DEGs
KOG	16 948	19
GO	6481	12
KEGG	9500	7
Swiss-Prot	12 427	25
nr	21 919	44
total	28 174	55

**Table 3.** Differentially expressed genes (DEGs) between virgin and mated females. Sign: FDR, false discovery rate; log_2_FC, log2 fold change.

number	gene ID	FDR	log_2_FC	GO	KOG	Swiss-Prot	nr	KEGG_pathway
1	c40539.graph_c0	1.44E−17	1.33	—	amino acid transport and metabolism	beta-ureidopropionase OS = *Dictyostelium discoideum* GN = pyd3 PE = 1 SV = 1	predicted: beta-ureidopropionase-like [*Nasonia vitripennis*]	pyrimidine metabolism (ko00240); beta-alanine metabolism (ko00410); pantothenate and CoA biosynthesis (ko00770); drug metabolism—other enzymes (ko00983)
2	c40539.graph_c1	4.04E−12	1.09	biological process: nitrogen compound metabolic process (GO:0006807); molecular function: hydrolase activity, acting on carbon-nitrogen (but not peptide) bonds (GO:0016810)	amino acid transport and metabolism	beta-ureidopropionase OS = *Dictyostelium discoideum* GN = pyd3 PE = 1 SV = 1	predicted: beta-ureidopropionase-like [*Nasonia vitripennis*]	pyrimidine metabolism (ko00240); beta-alanine metabolism (ko00410); pantothenate and CoA biosynthesis (ko00770); drug metabolism—other enzymes (ko00983)
3	c47555.graph_c0	5.59E−06	1.27	—	—	—	predicted: titin isoform X3 [*Nasonia vitripennis*]	—
4	c48536.graph_c0	0.001191	1.76	molecular function: catalytic activity (GO:0003824)	—	—	fatty acid synthase [*Bombyx mori*]	—
5	c47989.graph_c4	0.00995	1.34	—	—	probable cytochrome P450 4p2 OS = *Drosophila melanogaster* GN = Cyp4p2 PE = 2 SV = 1	predicted: cytochrome P450 4C1-like isoform X1 [*Nasonia vitripennis*]	—
6	c22148.graph_c0	2.39E−09	−5.20	—	—	ejaculatory bulb-specific protein 3 OS = *Drosophila melanogaster* GN = EbpIII PE = 2 SV = 2	predicted: ejaculatory bulb-specific protein 3-like [*Polistes dominula*]	—
7	c44788.graph_c0	3.28E−09	−1.08	molecular function: catalytic activity (GO:0003824); biological process: single-organism metabolic process (GO:0044710)	amino acid transport and metabolism	—	predicted: sarcosine dehydrogenase, mitochondrial [*Nasonia vitripennis*]	—
8	c46224.graph_c3	5.43E−08	−1.13	—	translation, ribosomal structure and biogenesis	excitatory amino acid transporter OS = *Caenorhabditis elegans* GN = glt-1 PE = 1 SV = 2	predicted: tRNA (adenine(58)-N(1))-methyltransferase non-catalytic subunit TRM6 [*Ceratosolen solmsi marchali*]	—
9	c30533.graph_c0	2.77E−07	−1.06	—	—	—	—	—
10	c37608.graph_c0	5.50E−07	−2.51	—	—	—	—	—
11	c32071.graph_c0	9.79E−07	−1.11	—	—	—	predicted: uncharacterized protein LOC105363533 [*Ceratosolen solmsi marchali*]	—
12	c33559.graph_c1	1.04E−06	−5.93	—	—	putative UDP-glucuronosyltransferase ugt-47 OS = *Caenorhabditis elegans* GN = ugt-47 PE = 1 SV = 2	predicted: UDP-glucuronosyltransferase 2A1-like [*Nasonia vitripennis*]	—
13	c43661.graph_c0	7.82E−06	−1.78	—	—	—	predicted: uncharacterized protein LOC100680050 isoform X1 [*Nasonia vitripennis*]	—
14	c46098.graph_c0	9.06E−06	−1.14	molecular function: DNA binding (GO:0003677); oxidoreductase activity (GO:0016491); cellular component: nucleus (GO:0005634); biological process: transcription initiation from RNA polymerase II promoter (GO:0006367); positive regulation of transcription, DNA-templated (GO:0045893); oxidation–reduction process (GO:0055114)	amino acid transport and metabolism	probable saccharopine dehydrogenase [NADP(+), l-glutamate-forming] OS = *Dictyostelium discoideum* GN = sdh PE = 2 SV = 1	predicted: alpha-aminoadipic semialdehyde synthase, mitochondrial isoform X1 [*Nasonia vitripennis*]	lysine degradation (ko00310)
15	c38071.graph_c0	9.06E−06	−1.40	—	—	—	predicted: RNA polymerase II degradation factor 1-like [*Trachymyrmex septentrionalis*]	—
16	c43170.graph_c1	9.08E−06	−1.26	molecular function: phosphoenolpyruvate carboxykinase (GTP) activity (GO:0004613); kinase activity (GO:0016301); GTP binding (GO:0005525); biological process: gluconeogenesis (GO:0006094); phosphorylation (GO:0016310)	energy production and conversion	phosphoenolpyruvate carboxykinase [GTP] OS = *Drosophila melanogaster* GN = Pepck PE = 2 SV = 2	predicted: phosphoenolpyruvate carboxykinase [GTP]-like [*Nasonia vitripennis*]	glycolysis/gluconeogenesis (ko00010); citrate cycle (TCA cycle) (ko00020); pyruvate metabolism (ko00620); FoxO signalling pathway (ko04068)
17	c49157.graph_c0	1.45E−05	−1.22	—	—	—	—	—
18	c38677.graph_c0	2.95E−05	−4.45	—	general function prediction only	glucose dehydrogenase [FAD, quinone] OS = *Drosophila pseudoobscura pseudoobscura* GN = Gld PE = 3 SV = 4	predicted: glucose dehydrogenase [FAD, quinone]-like [*Nasonia vitripennis*]	—
19	c28240.graph_c0	3.40E−05	−5.47	—	—	ejaculatory bulb-specific protein 3 OS = *Drosophila melanogaster* GN = EbpIII PE = 2 SV = 2	predicted: uncharacterized protein LOC100113667 [*Nasonia vitripennis*]	—
20	c48156.graph_c5	0.000103	−1.37	—	—	—	—	—
21	c40480.graph_c0	0.000118	−1.93	—	lipid transport and metabolism	retinol-binding protein pinta OS = *Drosophila melanogaster* GN = pinta PE = 2 SV = 1	predicted: alpha-tocopherol transfer protein-like [*Copidosoma floridanum*]	—
22	c47339.graph_c0	0.000149	−1.81	—	—	—	predicted: uncharacterized protein LOC100122494 [*Nasonia vitripennis*]	—
23	c46393.graph_c7	0.000169	−1.38	molecular function: phosphatidylinositol phospholipase C activity (GO:0004435); signal transducer activity (GO:0004871); guanyl-nucleotide exchange factor activity (GO:0005085); calcium ion binding (GO:0005509); cellular component: intracellular (GO:0005622); biological process: lipid metabolic process (GO:0006629); small GTPase mediated signal transduction (GO:0007264)	—	1-phosphatidylinositol 4,5-bisphosphate phosphodiesterase epsilon-1 OS = *Caenorhabditis elegans* GN = plc-1 PE = 1 SV = 1	predicted: 1-phosphatidylinositol 4,5-bisphosphate phosphodiesterase epsilon-1-like [*Nasonia vitripennis*]	inositol phosphate metabolism (ko00562); phosphatidylinositol signalling system (ko04070); AGE-RAGE signalling pathway in diabetic complications (ko04933)
24	c30180.graph_c0	0.000325	−1.41	—	—	—	venom protein N precursor [*Nasonia vitripennis*]	—
25	c37769.graph_c0	0.000522	−1.58	—	carbohydrate transport and metabolism	putative inorganic phosphate cotransporter OS = *Drosophila ananassae* GN = Picot PE = 3 SV = 1	predicted: sialin-like [*Ceratosolen solmsi marchali*]	—
26	c38035.graph_c1	0.000522	−2.21	—	—	—	predicted: uncharacterized protein LOC100113667 [*Nasonia vitripennis*]	—
27	c41154.graph_c0	0.000699	−5.36	—	—	—	predicted: general odorant-binding protein 56d [*Nasonia vitripennis*]	—
28	c32591.graph_c0	0.000883	−1.38	—	general function prediction only	opsin, blue-sensitive OS = *Apis mellifera* GN = BLOP PE = 1 SV = 2	predicted: opsin, blue-sensitive [*Nasonia vitripennis*]	—
29	c49861.graph_c0	0.000883	-Inf	molecular function: oxidoreductase activity, acting on CH-OH group of donors (GO:0016614); biological process: single-organism metabolic process (GO:0044710)	general function prediction only	glucose dehydrogenase [FAD, quinone] OS = *Drosophila pseudoobscura pseudoobscura* GN = Gld PE = 3 SV = 4	predicted: glucose dehydrogenase [FAD, quinone]-like [*Trichogramma pretiosum*]	—
30	c43486.graph_c0	0.00102	−3.61	—	amino acid transport and metabolism	chymotrypsin-2 OS = *Vespa crabro* PE = 1 SV = 1	serine protease 137 precursor [*Nasonia vitripennis*]	—
31	c44805.graph_c0	0.0011	−1.23	—	—	—	predicted: uncharacterized protein LOC100122494 [*Nasonia vitripennis*]	—
32	c48890.graph_c0	0.00117	−1.03	—	amino acid transport and metabolism	chymotrypsin-2 OS = *Vespa crabro* PE = 1 SV = 1	predicted: chymotrypsin-2-like [*Copidosoma floridanum*]	neuroactive ligand-receptor interaction (ko04080)
33	c30678.graph_c0	0.00128	−6.30	—	general function prediction only	venom carboxylesterase-6 OS = *Apis mellifera* PE = 2 SV = 1	carboxylesterase clade B, member 6 precursor [*Nasonia vitripennis*]	—
34	c22369.graph_c0	0.0013	−1.17	—	—	—	predicted: uncharacterized protein LOC100115024 [*Nasonia vitripennis*]	—
35	c45559.graph_c0	0.00173	−1.02	—	—	arylphorin subunit alpha OS = *Manduca sexta* PE = 2 SV = 1	predicted: hexamerin 70b isoform X1 [*Nasonia vitripennis*]	—
36	c29056.graph_c0	0.00179	−1.31	—	—	—	predicted: pheromone-binding protein Gp-9 [*Nasonia vitripennis*]	—
37	c21285.graph_c0	0.00185	−6.30	molecular function: odorant binding (GO:0005549)	—	general odorant-binding protein 83a OS = *Drosophila melanogaster* GN = Obp83a PE = 1 SV = 1	predicted: general odorant-binding protein 69a [*Nasonia vitripennis*]	—
38	c34718.graph_c0	0.00195	−2.09	—	—	—	—	—
39	c41422.graph_c0	0.00214	−1.32	cellular component: integral component of membrane (GO:0016021); molecular function: transmembrane transporter activity (GO:0022857); biological process: transmembrane transport (GO:0055085)	general function prediction only	carcinine transporter OS = *Drosophila melanogaster* GN = CarT PE = 2 SV = 1	predicted: carcinine transporter isoform X1 [*Ceratina calcarata*]	—
40	c33252.graph_c0	0.00234	−1.39	—	—	—	predicted: uncharacterized protein LOC106647010 [*Copidosoma floridanum*]	—
41	c37607.graph_c0	0.00234	−1.18	—	—	—	—	—
42	c48570.graph_c2	0.00246	−1.03	—	—	—	—	—
43	c47166.graph_c0	0.00247	−1.37	—	lipid transport and metabolism	1-phosphatidylinositol 4,5-bisphosphate phosphodiesterase OS = *Drosophila melanogaster* GN = norpA PE = 1 SV = 4	predicted: 1-phosphatidylinositol 4,5-bisphosphate phosphodiesterase-like [*Nasonia vitripennis*]	—
44	c43794.graph_c0	0.00314	−1.43	molecular function: hydrolase activity, hydrolyzing O-glycosyl compounds (GO:0004553); biological process: chitin metabolic process (GO:0006030)	carbohydrate transport and metabolism	probable chitinase 2 OS = *Drosophila melanogaster* GN = Cht2 PE = 1 SV = 1	predicted: chitotriosidase-1-like isoform X1 [*Nasonia vitripennis*]	amino sugar and nucleotide sugar metabolism (ko00520)
45	c44018.graph_c0	0.00318	−1.41	—	—	—	—	—
46	c48776.graph_c0	0.00319	−1.94	—	—	—	—	—
47	c46454.graph_c0	0.00328	−1.37	—	—	—	predicted: uncharacterized protein LOC108766667 [*Trachymyrmex cornetzi*]	—
48	c40631.graph_c0	0.00468	−1.06	—	—	—	predicted: uncharacterized protein LOC100680146 [*Nasonia vitripennis*]	—
49	c44319.graph_c1	0.00702	−1.24	—	general function prediction only	rhodopsin OS = *Camponotus atriceps* PE = 2 SV = 1	predicted: rhodopsin-like [*Nasonia vitripennis*]	—
50	c35460.graph_c0	0.00749	−1.22	—	—	—	—	—
51	c49077.graph_c0	0.00803	−1.58	—	—	—	predicted: serine protease inhibitor 3-like isoform X2 [*Bombus impatiens*]	—
52	c42015.graph_c0	0.00885	−1.49	biological process: intracellular signal transduction (GO:0035556)	—	—	predicted: uncharacterized protein LOC100678008 [*Nasonia vitripennis*]	—
53	c46871.graph_c1	0.00928	−1.31	—	—	—	—	—
54	c21488.graph_c0	0.00948	−1.37	cellular component: extracellular region (GO:0005576); biological process: chitin metabolic process (GO:0006030); molecular function: chitin binding (GO:0008061)	—	—	predicted: uncharacterized protein LOC100120615 [*Nasonia vitripennis*]	—
55	c41137.graph_c0	0.00995	−2.29	—	signal transduction mechanisms	probable serine/threonine-protein kinase DDB_G0270146 OS = *Dictyostelium discoideum* GN = DDB_G0270146 PE = 3 SV = 1	predicted: dual specificity protein kinase shkE-like [*Trichogramma pretiosum*]	—

In the GO enrichment analyses, subcategories were enriched among the downregulated genes in mated females, mainly involved in chitin metabolism (GO: 0006030; *p* = 0.005), phosphoenolpyruvate carboxykinase activity (GO:0004613; *p* = 0.048) and positive regulation of transcription, DNA-templated (GO:0045893; *p* = 0.048). Subcategories of hydrolase activity, acting on carbon–nitrogen (but not peptide) bonds (GO:0016810; *p* = 0.01) and nitrogen compound metabolic processes (GO:0006807; *p* = 0.004) were enriched among the upregulated genes in mated females.

### 3.2. Mating

**Figure 1.**
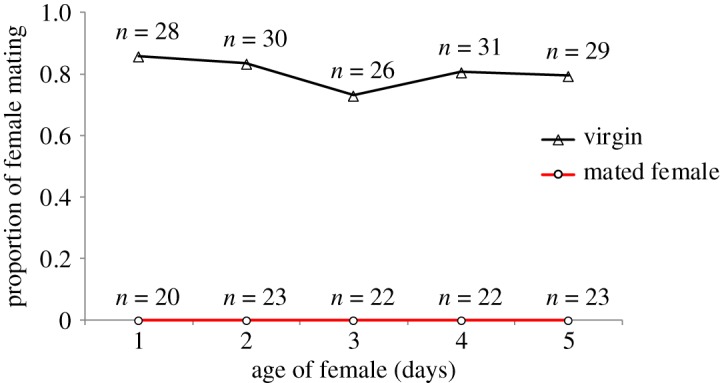
Mating capacity of mated and virgin females with different ages. Mating capacity was measured by the proportion of females successfully completed mating with male during 60 min.

When males were offered to virgins ranging from 1 to 5 days in age, approximately 80.42% of the virgins exhibited successful mating, which was not significantly affected by age (*χ*^2^ = 1.55, d.f. = 4, *p* > 0.05). Most of the mating behaviour occurred 10 min after a male was offered. After a virgin female mated with a male, she was not observed to mate again with the same male or another male. With increasing age, the mated females also ceased to exhibit mating behaviour (figure 1). Additionally, we observed that males still fan and run towards mated females as they do virgin females.

**Figure 2.**
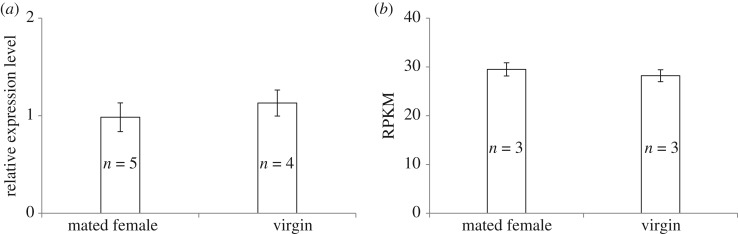
Expression of PBAN genes from qRT-PCR and RNASeq. (*a*) The expression of PBAN genes determined through qRT-PCR was calculated by the 2^−Δ^*^Δ^*^Ct^ method using the housekeeping gene EF1A as a reference to eliminate sample-to-sample variations in the initial cDNA samples. (*b*) The expression of PBAN genes determined through RNASeq was identified by the RPKM method.

The expression of the PBAN gene determined through qRT-PCR and RNASeq was calculated by the 2^−ΔΔCt^ and RPKM methods, respectively. Results showed that the expression of the PBAN gene was not significantly different between virgin and mated females (figure 2*a*: qRT-PCR, *t* = −0.71, d.f._1_ = 1, d.f._2_ = 7, *p* > 0.05; figure 2*b*: RPKM, FDR = 0.9997, log_2_FC = −^1^0.0308).

### 3.3. Effect of mating status on egg loading

**Figure 3.**
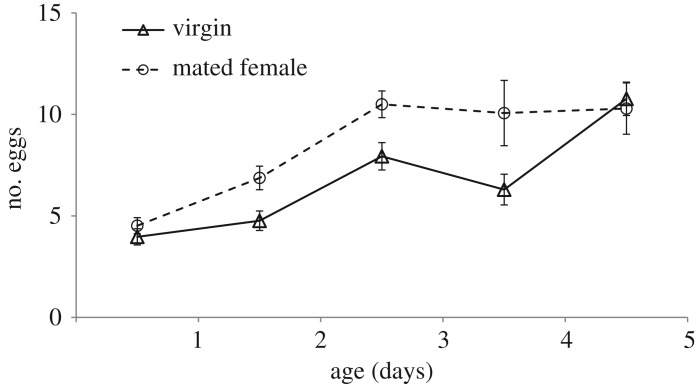
Mean egg loads (±SEs) over time of mated and unmated *Anastatus disparis* females. Egg loads were measured in terms of the number of mature eggs in the ovaries. And the age of measured females ranged from 1 to 5 days old.

After female eclosion, few mature eggs (virgin females: 3.97 ± 0.4; mated females: 4.52 ± 0.4) were observed in the ovaries. The number of mature eggs in virgin and mated females showed an increasing tendency with individual age (virgin females: *R*^2^ = 0.465, *p* = 0.000; mated females: *R*^2^ = 0.436, *p* = 0.000). The result of GLMM analysis showed that the number of mature eggs in the females was significantly influenced by individual age (*F* = 20.28, d.f._1_ = 4, d.f._2_ = 268, *p* = 0.000), and mated females loaded significantly more mature eggs than virgin females (*F* = 8.69, d.f._1_ = 1, d.f._2_ = 270, *p* = 0.003; figure 3). At day 5, the mature egg counts of the mated females (10.77 ± 0.82) and virgins (10.29 ± 1.26) were not significantly different (*p* > 0.05).

## 4. Discussion

Mating often induces behavioural and physiological changes in female insects [5]. In the egg parasitoid wasp *A. disparis*, post-mating females become unattractive and exhibit accelerated egg maturation (figures 1 and 3), which is accompanied by substantial changes in gene expression (table 3). A total of 55 DEGs were identified in post-mating females, and most (90.9%) of the DEGs were downregulated. Overall, the changes in gene expression prompted by mating observed in our study provide insight and useful information to improve comprehension of behavioural and physiological changes, which are discussed below.

With respect to the mode of egg production, parasitoids can be classified as pro-ovigenic or synovigenic. Pro-ovigenic species mature all or most of their lifetime complement of eggs prior to emergence from hosts, whereas synovigenic species emerge with very few or no mature eggs and have to mature the eggs as they age [48,49]. Therefore, *A. disparis* is thought to be a synovigenic species in which the number of eggs per day (figure 3, max. = 15) is significantly less than the lifetime fecundity of hundreds [23], and the number of mature eggs increases with female age. In addition, we found that as female age increased, the egg loads in mated females increased more rapidly than those in virgin females (figure 3). Gillott & Friedel [50] and Wheeler [51] reviewed ‘fecundity-enhancing substances' in addition to sperm that are transferred by male insects during mating and that stimulate oogenesis, egg maturation and oviposition. Our transcriptional data indicate that increased egg loads in mated females are associated with high expression of the fatty acid synthase (FASN) gene, which encodes the enzyme catalysing fatty acid synthesis [52–54] and is upregulated in mated females (table 3). *FAS* expression has been demonstrated to be related to fecundity in insects; in *Nilaparvata lugens*, when *FAS* expression decreased, female weights, ovarian total lipids and the number of oviposited eggs also significantly decreased [55]. A similar finding showed that *FAS* silencing suppressed fatty acid biosynthesis and decreased fecundity in the mosquito *Aedes aegypti* [56]. In addition, increased egg production in mated females might require that females allocate resources away from somatic maintenance and invest resources in reproductive processes [57], which may suggest that many genes related to metabolism exhibit changes after female mating according to our transcriptional data. For example, there were two upregulated genes involved in pyrimidine metabolism (c40539.graph_c0, c40539.graph_c1). While most of the genes were involved in the metabolism of lipids, carbohydrates and amino acids (e.g. c47166.graph_c0; c43794.graph_c0; c49861.graph_c0; c21488.graph_c0; c46393.graph_c7; c44788.graph_c0; table 3), hexamerin (c45559.graph_c0) was also downregulated, which may reflect a trade-off between reproductive and nonreproductive processes [58], likely because egg production is energetically costly and females shift from nutrient storage to utilization as their stores are depleted [59].

Polyandrous females can gain direct and indirect benefits [60–64]. Similar to many parasitoid wasp species (reviewed by Ridley [65]), the *A. disparis* females in this study also exhibited the characteristics of monandry, in which post-mating females rejected subsequent mating (figure 1). As male *A. disparis* mate only once, they can supply females with sufficient sperm for subsequent reproduction [23]; thus, females may refuse to mate multiple times to avoid wasting time and energy. Several studies have shown that females of some parasitoid species may re-mate if they have mated with sperm-depleted males [16,66], which will be studied further. Furthermore, during copulation, males can transfer certain chemicals with the spermatozoa [67,68], which may include toxic compounds, such as those found in *Drosophila* fruit flies [69], the bruchid *Acanthoscelides obtectus* [70] and the nematode *Caenorhabditis elegans* [71]. Other negative effects of multiple mating include concomitant increased vulnerability to predation, sexual diseases, parasites and pathogens [72,73]. A cytochrome P450 gene (c47989.graph_c4) was found to be upregulated by mating in females, which may be involved in detoxification [74]. In addition, the post-mating expression levels of four protease genes change, among which predicted serine protease genes (c43486.graph_c0) and a chymotrypsin gene (c48890.graph_c0) were downregulated after mating. Induced proteases in virgin female could protect females from harmful proteins introduced during mating [58]. Females receive sperm from their mates, then maintain the sperm in storage organs to await opportunities for fertilization. A serine protease inhibitor (c49077.graph_c0) was downregulated after mating, which may play a role in protecting sperm from degradation or expose sperm surface proteins needed for storage or fertilization [58].

Females may cease to attract males after mating resulting in mating only once [75]. It has been shown that mating generally causes changes in attractiveness in many species of moths and parasitoids, which are correlated with pheromone levels [41,77,76]. However, as shown in *Spalangia endius* [78], we observed that males fan and run towards mated females, and our q-PCR results (figure 2*a*) and transcriptional analyses (figure 2*b*) showed that expression of the PBAN gene was not significantly different between virgin and mated females. This finding suggested that because the production of attractants may not cease or decrease after mating, mating might not cause changes in the attractiveness of females to males, and the mating of *A. disparis* females only once may therefore be unlikely to be caused by lower attractiveness of mated females (also see *Cotesia flavipes* [79]). Besides, odorant-binding proteins (OBPs) are a class of olfactory proteins and are thought to aid in the capture and transport of odorants and pheromones to receptors [80]. In fruit flies, OBP expression levels in females changed significantly after mating [81], and ectopic expression of Obp99b in female fat body tissue led to reduced receptivity and mating success [82]. Our transcriptome data showed that a total of three annotated genes associated with OBPs were downregulated in *A. disparis* females after mating (table 3; c41154.graph_c0; c29056.graph_c0; c21285.graph_c0), which may explain why mated females become unreceptive to further mating. In addition, our transcriptional data also showed that four genes (c40480.graph_c0; c41422.graph_c0; c44319.graph_c1; c32591.graph_c0) that are expressed in the adult eye or are known to function in visual transduction (including opsin, rhodopsin and carcinine transporter [59,83]; table 3) were downregulated after mating (see also the honeybee and *Apis florae* [84,85]). Altered expression of vision genes could impact a female's response to other females or males [83]. Therefore, the downregulation of vision-related genes after mating in our species may also influence the re-mating behaviour of females. Rather than a change in female pheromone related attractive, our results suggested that decreased visual and odorant-binding abilities also resulted in mated females becoming unreceptive and refusing to mate again.

In addition, as shown in other studies [58,59], other genes in our study, for example, involved in chitin metabolism (c43794.graph_c0; c21488.graph_c0), signal transduction (c41137.graph_c0; c42015.graph_c0), that exhibit ectopic expression after mating involved in post-mating behavioural and physiological responses, while those with unknown or unclear function require further study. By identifying changes in gene expression prompted by mating, our study provided new insights into changes in behavioural and physiological aspects. Simultaneously, this dataset provides a basis for future mechanistic studies examining how specific genes mediate behavioural and physiological changes in females post-mating. Additionally, understanding how these changes in gene expression orchestrate the post-mating response in this species may provide insight into the reproductive behaviour of more complex animals.

## Supplementary Material

Statistics of transcriptome assembly and predicted unigenes

## Data accessibility

The datasets supporting this article have been uploaded as part of the electronic supplementary material. All raw sequence data files are available from the Sequence Read Archive database (accession no. PRJNA505044).
