# Supplementary material for: Behavioural and transcriptional changes in post-mating females of an egg parasitoid wasp species
Source: R Soc Open Sci. 2019 Jan 23;6(1):181453. doi: 10.1098/rsos.181453 (PMC6366167; doi:10.1098/rsos.181453)
Supplement: Statistics of transcriptome assembly and predicted unigenes [file rsos181453supp1.docx]

**Table S1.** Statistics of transcriptome assembly and predicted unigenes

| Length Range | Transcripts | Unigenes |
| --- | --- | --- |
| <300 bp | 0(0%) | 0(0%) |
| 300-500 bp | 33,896(25.57%) | 28,352(49.61%) |
| 500-1000 bp | 25,436(19.19%) | 15,948(27.90%) |
| 1000-2000 bp | 20,254(15.28%) | 6,212(10.87%) |
| >2000 bp | 52,957(39.95%) | 6,640(11.62%) |
|  |  |  |
| Total Number | 132,543 | 57,152 |
| Size of data (bp) | 332,731,541 | 59,690,235 |
| N50 length (bp) | 5,020 | 1,935 |
| Mean length (bp) | 2510.37 | 1044.41 |
